# Supplementary material for: Reconciling Mining with the Conservation of Cave Biodiversity: A Quantitative Baseline to Help Establish Conservation Priorities
Source: PLoS One. 2016 Dec 20;11(12):e0168348. doi: 10.1371/journal.pone.0168348 (PMC5173368; doi:10.1371/journal.pone.0168348)
Supplement: S1 Dataset — (ZIP) [file pone.0168348.s002.zip › Taxa/Serra Norte/SN_2013/N4E/Lista N4E-52.pdf]

CAVIDADE N4E-0052

Classe	Ordem	Fam/Outros	Gên/Outros	Espécie	S-U	U-S	Zon.
Arachnida	Acari	Laelapidae		sp.41	X		E
Arachnida	Amblypygi	Charinidae	*Charinus*	sp.1		1	E
Arachnida	Araneae	Filistatidae		sp.1	X		E
Arachnida	Araneae	Pholcidae	*Leptopholcus*	sp.1		X	E
Arachnida	Araneae	Pholcidae	*Mesabolivar*	aff. *cambridgei*	X		E
Arachnida	Araneae	Pholcidae	*Mesabolivar*	sp.1		X	E
Arachnida	Araneae	Symphytognathidae	*Anapistula*	sp.1	X		E
Arachnida	Araneae	Theridiosomatidae		jovem		X	E
Arachnida	Pseudoscorpiones	Chthoniidae		jovem	X		E
Diplopoda	Polydesmida	Fuhrmannodesmidae		sp.1		X	E
Diplopoda	Spirostreptida	Pseudonannolenidae	*Pseudonannolene*	jovem	2		E
Entognatha	Collembola	Cyphoderidae		sp.1	X		E
Entognatha	Collembola	Paronellidae		sp.1		X	E
Entognatha	Diplura	Projapygidae		sp.1		X	E
Insecta	Coleoptera	Dytiscidae		sp.11B	X		E
Insecta	Coleoptera	Dytiscidae		sp.12A	X		E
Insecta	Coleoptera	Staphylinidae	Pselaphinae	sp.12	X		E
Insecta	Coleoptera			jovem		X	E
Insecta	Diptera	Aulacigastridae		sp.		X	E
Insecta	Diptera	Cecidomyiidae	Cecidomyiinae	sp.	X		E
Insecta	Diptera	Psychodidae	*Breviscapus*	sp.		X	E
Insecta	Diptera	Psychodidae	Phlebotominae	sp.	X	X	E
Insecta	Diptera	Simuliidae		sp.	X		E
Insecta	Diptera	Tipulidae	Tipulinae	sp.		X	E
Insecta	Heteroptera	Reduviidae	Reduviinae	sp.		1	E
Insecta	Heteroptera	Tingidae		sp.	X		E
Insecta	Homoptera	Cixiidae		sp.2		X	E
Insecta	Homoptera	Cixiidae		sp.3	X		E
Insecta	Homoptera	Cixiidae		sp.4		X	E
Insecta	Hymenoptera	Formicidae	*Camponotus*	sp.1		X	E
Insecta	Hymenoptera	Formicidae	*Camponotus*	sp.2	X	X	E
Insecta	Hymenoptera	Formicidae	*Crematogaster*	sp.1		X	E
Insecta	Hymenoptera	Formicidae	*Dolichoderus*	aff. *bispinosus*	X		E
Insecta	Hymenoptera	Formicidae	*Gnamptogenys*	sp.1		X	E
Insecta	Hymenoptera	Formicidae	*Pheidole*	sp.1	X	X	E
Insecta	Hymenoptera	Formicidae	*Pheidole*	sp.2		X	E
Insecta	Hymenoptera	Formicidae	*Pheidole*	sp.3	X		E
Insecta	Lepidoptera	Tineoidea		sp.1	X	X	E
Insecta	Orthoptera	Gryllidae	Nemobiinae	sp.		1	E
Insecta	Orthoptera	Phalangopsidae	*Paraclodes*	sp.	3	12	E/P
Insecta	Psocoptera	Epipsocidae	*Epipsocus*	sp.2	X		E
Insecta	Psocoptera	Epipsocidae	*Mesepipsocus*	sp.1		X	E
Gastropoda	Pulmonata	Subulinidae		sp.5		X	E
Malacostraca	Isopoda	Armadillidae		sp.1		X	E
Turbellaria	Tricladida			sp.2		1	E
Amphibia	Anura	Strabomantidae	*Pristimantis*	cf. *fenestratus*	2		P
Mammalia	Chiroptera	Emballonuridae	*Peropteryx*	*kappleri*	3	3	P
